# Effects of hCG administration on accessory corpus luteum formation and progesterone production in estrous-induced nulliparous Santa Inês ewes

**DOI:** 10.21451/1984-3143-AR2017-957

**Published:** 2018-08-16

**Authors:** Jeferson Ferreira da Fonseca, Ana Carolina Rosa Castro, Eduardo Kenji Nunes Arashiro, Maria Emilia Franco Oliveira, Fabiana Nunes Zambrini, Luciana Vieira Esteves, Felipe Zandonadi Brandão, Joanna Maia Gonçalves Souza-Fabjan

**Affiliations:** 1 Embrapa Goats and Sheep Research Center, Estrada Sobral/Groaíras, Sobral, CE, Brazil.; 2 Faculty of Veterinary Medicine, Fluminense Federal University, Niterói, RJ, Brazil.; 3 Department of Preventative Veterinary Medicine and Animal Reproduction, School of Agricultural and Veterinarian Sciences, São Paulo State University (Unesp), Jaboticabal, São Paulo, Brazil.; 4 Department of Veterinary Medicine, Federal University of Viçosa, Av PH Rolfs s/n, Viçosa, MG, Brazil.

**Keywords:** CL, luteotropic effect, ovulation induction, progesterone, sheep, ultrasound

## Abstract

The effect of hCG administration on accessory corpus luteum (ACL) formation, CL area, and plasma progesterone (P4) concentration (ng/mL) seven days after breeding was studied in nulliparous Santa Inês sheep. Intravaginal 60 mg MAP sponges were inserted into ewes for six days and 300 IU eCG i.m. and 30 µg d-cloprostenol latero-vulvar were administered 24 h before sponge removal. Ewes were naturally bred and, seven days after first mating (Day 0; D0), were treated with either 250 IU hCG (hCG group; n = 7) or 1 mL saline solution (control group; n = 7). Blood was collected to determine plasma P4 concentrations and sonograms were performed on Days 7, 10, 13, 16, 19, and 22. Number of CL on D7 was similar (P > 0.05) between hCG (1.3 ± 0.5) and control (1.3 ± 0.5) groups; however, on D13, it was greater (P < 0.05) in the hCG group (2.3 ± 0.5) than in the control group (1.3 ± 0.5). A greater (P < 0.05) luteal tissue area was detected in hCG-treated ewes (n = 4) on Days 16 to 22 than in the animals in the control group (n = 7). Plasma P4 concentration on D13 to D22 was higher (P < 0.05) in hCG-treated animals than in control ewes. Administration of hCG seven days after estrus onset efficiently induced accessory CL formation in ewes, increasing luteal tissue area and plasma P4 concentration.

## Introduction

The Santa Inês sheep is considered to be the most diffused naturalized breed in Brazil. Originally from the Northeast region of the country, Santa Inês sheep can be both crossbreed and purebred stock, and they are now commonly found in the southeast region of Brazil. Geographical, seasonal, and climatic variations can directly impact the reproductive performance of this breed of sheep ( Balaro *et al.*, 2014 ; Oliveira *et al.*, 2016 ). In addition to marking a reduction or cessation of spontaneous estrus behavior, the non-breeding season is also associated with a decrease in plasma progesterone concentration in estrus-induced ewes in comparison to animals with natural estrus and breeding ( Rhind *et al.*, 1978 ; Wheeler and Barnes, 1983 ; Theodosiadou *et al*., 2004 ).

The positive effect of progesterone (P4) on embryo quality and its early development, uterine environment, and the reduction of pregnancy loss has been previously reported in ruminants (Wiltbank *et al.*, 1994; Lonergan *et al.*, 2016 ). The administration of hCG or other ovulation inductor (e.g., GnRH or LH) to induce accessory corpus luteum (ACL) formation and increased P4 concentration has been investigated in bovine ( Fonseca *et al.*, 2000 ; Fonseca *et al.*, 2001a , b ) and goats (Fonseca *et al.*, 2005; Fonseca *et al.*, 2006 ) with the objective of improving pregnancy rates. In a study on subtropical sheep, hCG treatment on Days 5 and 7.5 after estrus increased luteal weight and induced conversion of small luteal cells into large luteal cells, leading to a higher serum P4 concentration ( Farin *et al.*, 1988 ). However, the effects of hCG on ovary and P4 levels when administered to Santa Inês sheep in tropical conditions have not yet been investigated in depth and no study has evaluated the use of this drug to improve the reproductive performance of the Santa Inês breed in the southeast of Brazil, when ewes are bred outside the natural breeding season, which runs from September to December in this region ( Balaro et al., 2014 ). Thus, the objective of this study was to evaluate the effect the administration of hCG seven days after breeding on accessory corpus luteum formation, luteal tissue area and plasma P4 concentration in nulliparous Santa Inês ewes.

## Materials and Methods

### Experimental animals and facilities

This research was reviewed and approved by the Animal Care Committee of Embrapa Dairy Cattle (Protocol 15/2014) and conducted across two trials during the nonbreeding season of Santa Inês ( Balaro *et al*., 2014 ), between October and November, at the Experimental Campus of Embrapa Dairy Cattle, in the rural area of Coronel Pacheco, Brazil (latitude 21° 35’ S, longitude 43°15’ W, and altitude of 435 m). A total of 14 nulliparous Santa Inês ewes aged between 12 and 14 months were kept in an intensive system, and were fed corn silage and Napier grass (* Pennisetum purpureum v. Taiwan*) as forage. They were also administered with a balanced concentrate supplement according to their nutritional needs (National Research Council, 2007). Mineralized salt and drinking water were available to the ewes *ad libitum *.

### Hormonal protocol for estrus induction and hCG treatment

Estrus was induced using intravaginal sponges impregnated with 60 mg of medroxyprogesterone acetate (Progespon^®^, Syntex S.A., Buenos Aires, Argentina), which were inserted on a random day of the estrous cycle and left in place for six days. One day before sponge removal, all ewes received 300 IU eCG i.m. (Novormon^®^, Syntex S.A.) and latero-vulvar injection ( Fonseca *et al*., 2017 ) of 30 μg d-cloprostenol (Prolise^®^, ARSA S.R.L., Buenos Aires, Argentina). After sponge removal, estrus detection was performed twice a day using two fertile rams. After estrus onset, ewes were mated twice a day throughout the estrus period. The first day of mating was Day 0 (D0). On D7, ewes were randomly allocated into one of two treatment groups: hCG ewes (n = 7, 39.36 ± 0.64 kg and 3.14 ± 0.07 of BCS) received 250IU of hCG i.m. (Vetecor^®^, Hertape-Calier do Brasil Ltda, São Paulo, Brazil), while the control ewes (n = 7; 39.24 ± 0.52 kg and 3.25 ± 0.13 of BCS) received the same volume (1 mL) of saline solution i.m.

### Ovarian ultrasonography and luteal evaluation

Transrectal ultrasonography (US) exams were performed on Days 7, 10, 13, 16, 19, and 22 using a portable device equipped with a 7.5 MHz transducer (M5 Vet^®^, Mindray Medical International Limited, Shenzhen, China). Original CL was defined as CL that formed after ovulation associated with the onset of estrus. ACL was defined as CL that was not been detected on Day 7 of the estrous cycle but was identified on Day 13 of the cycle. The CL, ACL, and luteal tissue area (cm^2^) were determined using US equipment calipers. The luteal tissue area, which was estimated according to the largest diameter of each CL, was considered the sum of the area of all CLs detected in each animal. When present, the luteal cavity area was subtracted. The same experienced technician performed all the US exams and the equipment parameters (focus field, proximal, distal, and total gain) were standardized and maintained throughout the experiment period.

### Blood sampling and plasma P4 measurement

Before each US exam, blood samples were collected via jugular vein puncture and stored in tubes containing sodium EDTA within a vacuum system. Samples were centrifuged at 1000 *g* for 15 min at 5ºC. Plasma samples were then aspirated and stored at -20ºC in 1.5 mL tubes until plasma P4 determination. Plasma P4 concentration (ng/mL) was determined by radioimmunoassay (RIA), using commercial RIA kits (Beckman Coulter; Immunotech, Marseille, France). The assay sensitivity was 0.05 ng/mL. The mean intra- and inter-assay coefficient of variation was 12% and 9% respectively. In addition, all data were within the maximum and minimum points of the curve.

### Pregnancy diagnosis

Pregnancy rates were accessed by transrectal ultrasonography with the same equipment and by technician previous cited at 60 days after mating.

### Statistics and data analyses

Number of CL, luteal tissue area, and plasma P4 concentrations were analyzed for the main effect of treatment and days of estrous cycle (within each group) by one-way ANOVA. Differences between means were determined by Tukey’s test. A p-value less than 0.05 indicated that the difference was significant. Results are reported as mean ± SEM. All statistical analyses were performed using the System for Statistical Analysis (SAEG) software (Ribeiro Júnior, 2000).

## Results

After sponge removal, estrus behavior was observed in 85.7% (6/7) and 100% (7/7) of hCG and control animals, respectively. Regardless of the estrus response, all females in both groups were treated. At Day 7, US exam showed two ewes from each group with two CL, while the other animals had only one CL. The average number of CL at D7 was similar (P > 0.05) between hCG- (1.3 ± 0.5) and saline- (1.3 ± 0.5) treated ewes. At D13, hCG-treated ewes (2.3 ± 0.5) had more CLs (P < 0.05) than saline-treated (1.3 ± 0.5) ewes. None of the animals from the control group developed ACL. In the hCG group, the ACL formation rate was 85.7% (6/7), with five ewes developing one ACL and one ewe developing two ACL. The hCG-treated ewe that did not respond to the synchronization treatment had two CL at D7.

**Figure 1:**
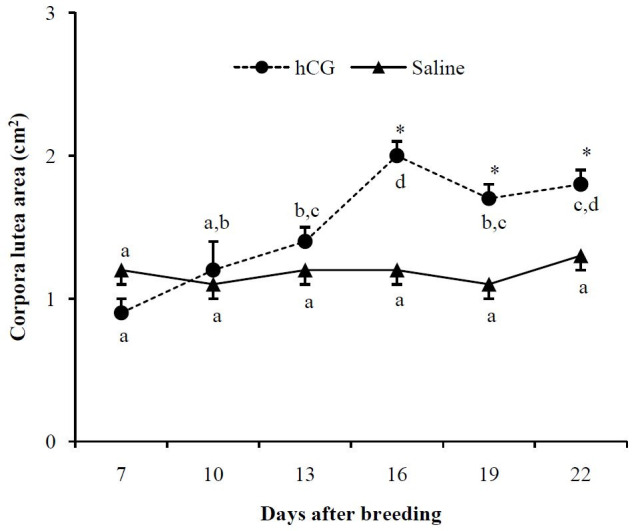
Corpora lutea area (cm^2^) in estrus induced nulliparous Santa Inês ewes receiving 250 IU hCG (1 mL) or saline (1 mL) intramuscularly at Day 7 after onset of estrus. * Indicate significant differences between groups in the respective day (Tukey test; P < 0.05). ^a,b,c,d^ Letters within groups indicate difference among days (Tukey test; P < 0.05).

Data regarding luteal tissue area and plasma progesterone concentration of non-pregnant ewes were not considered for comparison between groups. Luteal tissue area in control group did not change between D7 and D22 ( Fig. 1 ). Within the hCG group, however, the luteal tissue area progressively increased (P < 0.05) after hCG treatment until D16. A comparison of the two groups from D16 to D22 revealed that the luteal tissue area was greater (P < 0.05) in the hCG group than it was in the control group ( Fig. 1 ).

**Figure 2:**
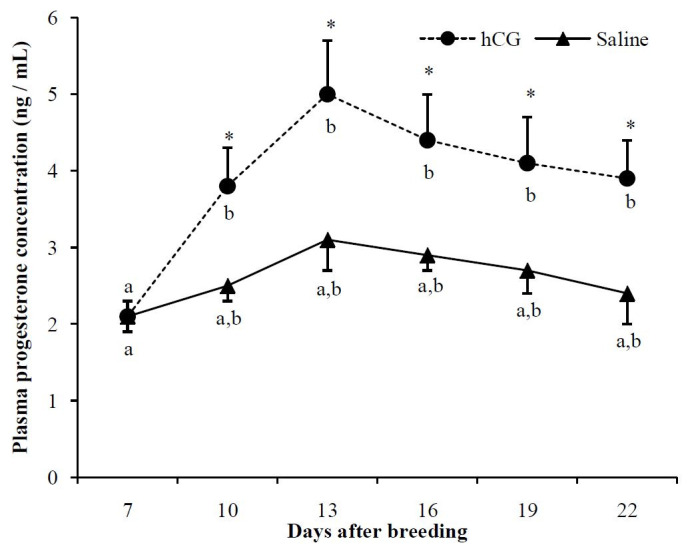
Plasma progesterone concentration (ng/mL) in estrus induced nulliparous Santa Inês ewes receiving 250 IU hCG (1 mL) or saline (1 mL) intramuscularly at Day 7 after onset of estrus. * Indicate significant differences between groups in the respective day (Tukey test; P < 0.05). ^a,b,c,d^ Letters within groups indicate difference among days (Tukey test; P < 0.05).

Plasma P4 concentration also exhibited changes over time. No significant changes (P > 0.05) in plasma P4 concentration in the control group were observed between D7 and D22. Plasma P4 concentration progressively increased (P > 0.05) within hCG-treated ewes until D10; however, no significant increase (P > 0.05) was observed in the subsequent days ( Fig. 2 ). A comparison between groups found that plasma P4 concentration was higher (P < 0.05) in the hCG-treated ewes than it was in the control ewes from D10 to D22.

Pregnancy rates, as confirmed on Day 60 post-mating, were 100% (7/7) and 66.7% (4/6) for saline- and hCG-treated ewes respectively.

## Discussion

The protocol of estrus induction resulted in 92.8% (13/14) estrus response. In the present study, administering 250 IU of hCG seven days after breeding efficiently induced the formation of ACL in estrus-induced nulliparous Santa Inês ewes, including the one that not show estrus after device removal. The Santa Inês breed appeared to represent a good model for this type of study since the Santa Inês sheep is a breed of relative lower prolificacy (1.3; Mexia *et al.*, 2004 ) and ovulation rate (1 to 1.3 ovulation per estrus induced ewe; Cavalcanti *et al.*, 2012 ; Teixeira *et al.*, 2016 ). Previous studies on the Santa Inês breed found that the number of follicular waves ranged from 2 to 5 with most animals presenting 4 follicular waves per cycle ( Oliveira *et al.*, 2016 ). Previous study found that ovulation in estrus-induced Santa Inês ewes occurred at around 24 h after estrus onset ( Cavalcanti *et al.*, 2012 ) and that the first follicular wave emerged near to ovulation. Thus, although daily ovary scanning was not performed, there is a possibility that hCG acted on dominant follicles during the first follicular wave (Day 6 after ovulation) evoking accessory ovulation. No differences in CL diameter, blood flow, and P4 concentration were reported between pregnant and non-pregnant cows until Day 6 following ovulation in a previous study ( Tarso *et al.*, 2016 ). In the present study, similar conditions were noted in Santa Inês sheep. Thus, any changes in the parameters studied took place following hormonal administration (seven days after breeding) and were probably evoked by exogenous hCG.

Because of CL detection at day 7, the ewe that did not show estrus also received hCG and formed ACL. It was probably a case of silent estrus. In the hCG group, the ACL formation rate was 85.7% (6/7) with one of these six ewes developing 2 ACL. This finding corroborates with a previous study performed in Western Range ewes. This study demonstrated that two doses of 300 IU hCG (at 5 and 7.5 days after estrus) induced the formation of 1 or 2 ACL ( Farin *et al.*, 1988 ). When short-term protocols were used for estrus synchronization/induction in sheep, ovulation occurred, on average, 60 h after sponge removal or 24 h after estrus onset ( Cavalcanti *et al.*, 2012 ). Therefore, in the present study, hCG treatment was administered 5-6 days after ovulation, when dominant follicles from the first follicular wave were already present in the ovaries and responsive to ovulation induction hormones ( Menchaca and Rubianes, 2004 ). It is also likely that the increase in P4 levels may alter the following follicular waves; however, this is a hypothesis that remains to be tested.

The formation of ACL observed in the present study increased the luteal tissue area within hCG-treated animals. This progressive increase in luteal tissue area was subsequently followed by a progressive increase in plasma P4 concentration. Previous studies on domestic ruminants demonstrated a significant positive correlation between luteal tissue area and plasma P4 concentration ( Siqueira *et al.*, 2009 ; Arashiro *et al.*, 2010 ; Figueira *et al.*, 2015 ). In addition, studies have also demonstrated that animals with multiple ovulations and, consequently, greater luteal tissue area, did not exhibit higher plasma P4 progesterone than animals with single ovulation ( Bartlewski *et al.*, 1999 ; Mann *et al.*, 2007 ; Arashiro *et al.*, 2010 ; Figueira *et al.*, 2015 ). However, in all these studies, multiple ovulations occurred naturally; i.e., without any exogenous hormonal treatment. In sheep, it was demonstrated that hCG treatment resulted in the conversion of small luteal cells to large luteal cells ( Farin *et al.*, 1988 ), thereby increasing P4 synthesis. Thus, it is likely that the increase in plasma P4 concentration observed in the hCG group was not only caused by the increase in the luteal tissue area, but also by the luteotrophic effect of hCG.

In hCG-treated ewes, a temporal difference was observed between the increase in luteal tissue area and plasma P4 concentration. The progressive increase in plasma P4 progesterone reached a plateau phase earlier than the increase in luteal tissue area (D10 and D16 respectively). This same pattern was observed when these parameters were compared between groups. During the natural estrous cycle, the luteal tissue area increased faster than plasma P4 progesterone in bovine ( Siqueira *et al.*, 2009 ), while both parameters reached the plateau phase at the same time in goats ( Arashiro *et al.*, 2010 ). In sheep, plasma P4 concentration increased faster than luteal tissue area during natural estrous cycle ( Figueira *et al.*, 2015 ); however, the temporal difference was only 2 days, significantly less time than that observed in the present study (6 days). This finding demonstrated that the luteotrophic effect of hCG accelerated the functional maturation of luteal tissue, leading to a more rapid increase in plasma P4 concentration.

Thus, plasma P4 concentration was significantly higher in hCG-treated ewes than it was in the control ewes before the expected moment of luteolysis. One of the causes of embryonic loss in mammals is inadequate plasma progesterone concentration in the critical window of maternal recognition of pregnancy. A series of studies demonstrated a positive relationship between early and mid-luteal phase concentrations of progesterone and subsequent embryo survival rate (reviewed by Diskin and Morris, 2008 ). In addition, research has found that, when the estrous cycle was induced in ewes, plasma P4 concentration was 70% lower than it was in animals with natural estrus and breeding cycles ( Rhind *et al.*, 1978 ; Theodosiadou *et al.*, 2004 ). One study found that the administration of hCG on Days 11, 12, and 13 after breeding resulted in significantly higher pregnancy rates in treated ewes (58%) than in non-treated (29%) ewes ( Kittok *et al.*, 1983 ). Thus, administering hCG on Day 7 of the estrus cycle as a means of increasing accessory CL formation, as tested in the present study, could be an effective method by which it is possible to increase P4, and potentially pregnancy rate, when progesterone is the main limiting and maintaining factor for pregnancy.

Finally, due to the limited number of ewes used in the present study (seven ewes per group), it was not possible to form definitive conclusions pertaining to conception rate. Further studies in field conditions with more animals are necessary to confirm the extent to which hCG administration influences CL function and, subsequently, conception rate within this breed of ewes.

## Conclusion

The results of the present study showed that the administration of 250 IU hCG seven days after estrus onset efficiently induced ACL formation in nulliparous Santa Inês ewes. This ACL formation leads to a significant increase in luteal tissue area and plasma progesterone concentration.
